# Extracorporeal blood filtration leading to tumor growth arrest and reduced analgesic requirements in Stage IV poorly differentiated pancreatic adenocarcinoma: A case report

**DOI:** 10.18632/oncotarget.28756

**Published:** 2025-07-23

**Authors:** Susanna Ulahannan, Peyton Smith, Jennifer Rios, Lakhmir Chawla

**Affiliations:** ^1^Stephenson Cancer Center, University of Oklahoma Health Sciences Center, OK 73104, USA; ^2^ExThera Medical, Martinez, CA 94553, USA; ^3^Department of Medicine, Veteran Affairs Medical Center, San Diego, CA 92121, USA

**Keywords:** extracorporeal blood filtration, circulating tumor cells, metastatic pancreatic cancer, seraph 100, OncoBind

## Abstract

Background: Despite significant strides in the management of metastatic solid tumors over the past few decades, metastatic disease remains a major clinical challenge, often leading to unfavorable patient outcomes. Circulating tumor cells (CTCs), which shed from the primary tumor, have the potential to disseminate and establish distant metastases, contributing to disease progression and reduced survival rates. Removal of CTCs via extracorporeal blood filtration could have significant therapeutic implications.

Case: A 51-year-old woman was diagnosed with metastatic poorly differentiated adenocarcinoma after presenting with severe abdominal pain. She deferred conventional chemotherapy options and elected treatment with CTC removal using an extracorporeal blood filter. After 9–12 filtration sessions of treatment over 12 months, she reported significant clinical improvement and staging scans demonstrated stable disease without evidence of new metastases.

Conclusion: Therapeutic modalities that explore CTC removal via blood filtration may potentially have promising clinical benefits. More prospective studies are required to determine the utility of this therapeutic strategy in patients with metastatic solid tumors. Our patient demonstrated significant clinical improvement with scans demonstrating stable disease.

## INTRODUCTION

Metastatic disease remains a formidable challenge in oncologic management, often associated with poor prognosis and significantly reduced quality of life, underscoring the critical need for improved therapeutic strategies. Circulating tumor cells (CTC’s) are tumor cells that are shed from the primary tumor and intravasate into peripheral blood, facilitating distant organ metastasis [1]. The *Seraph*® *100 Microbind*® *Affinity Blood Filter* (ExThera Medical, Martinez, CA – Seraph) is a broad-spectrum sorbent extracorporeal hemoperfusion device intended for reduction of sepsis mediating pathogens from the bloodstream. Recently, there has been interest in CTC removal using Seraph 100 to determine therapeutic benefits from high-yield CTC removal in metastatic or potentially metastatic cancer. In *ex vivo* studies, endpoint attached heparin surfaces have been shown to remove circulating tumor cells and microvesicles [2, 3]. Seraph 100 has been shown to remove CTCs in a preliminary study of patients with metastatic disease [4]. Seraph may also alter the levels of other circulating factors in blood (e.g., cytokines, coagulation factors) providing additional therapeutic benefit. Here, we report the case of a patient with Stage IV poorly differentiated pancreatic adenocarcinoma who was treated with Seraph, and we report single patient safety, potential therapeutic benefit, and changes in circulating factors such as CTCs.

## CASE

**Figure 1:**
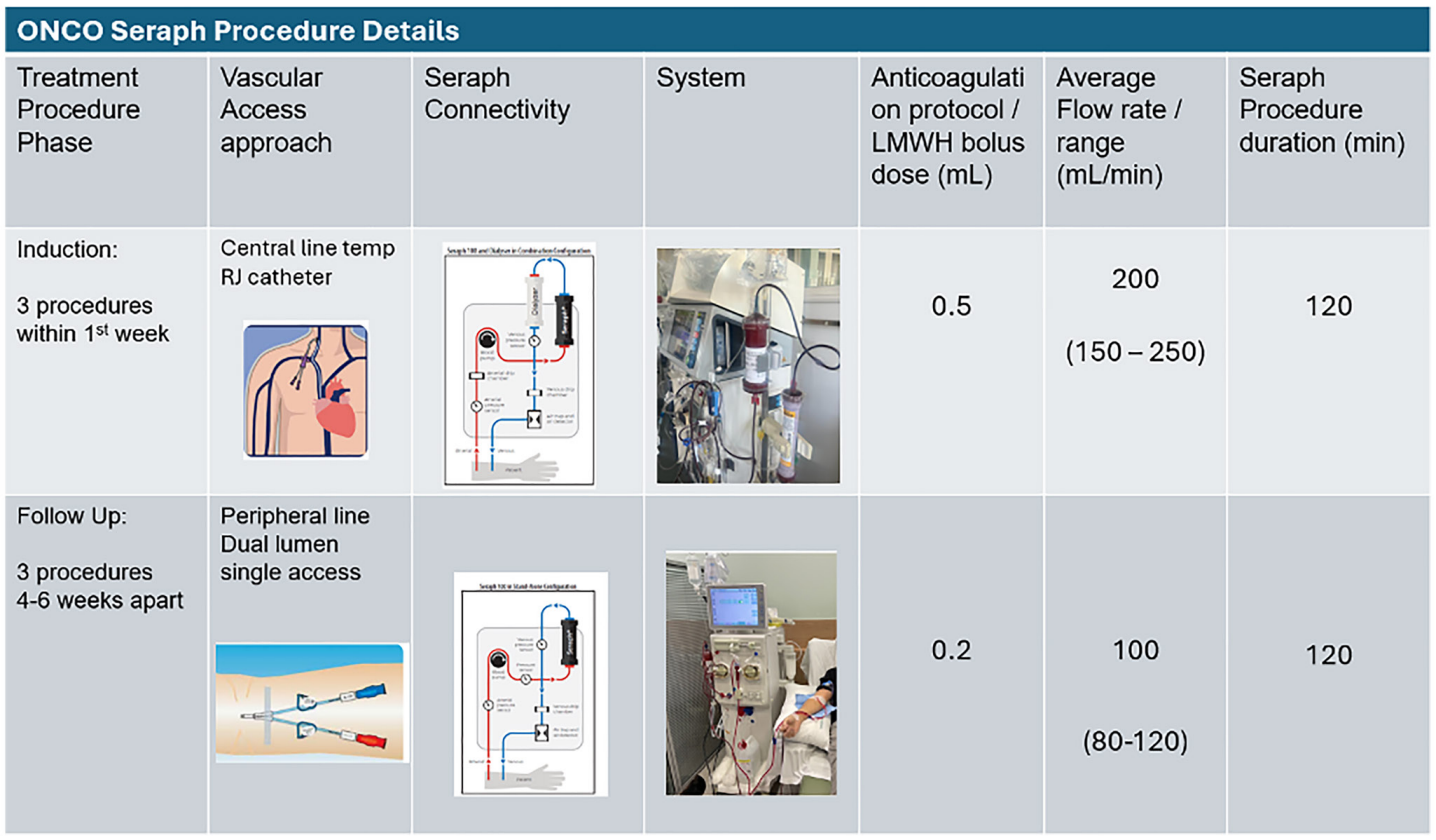
Summary of extracorporeal blood filtration procedure.

**Figure 2:**
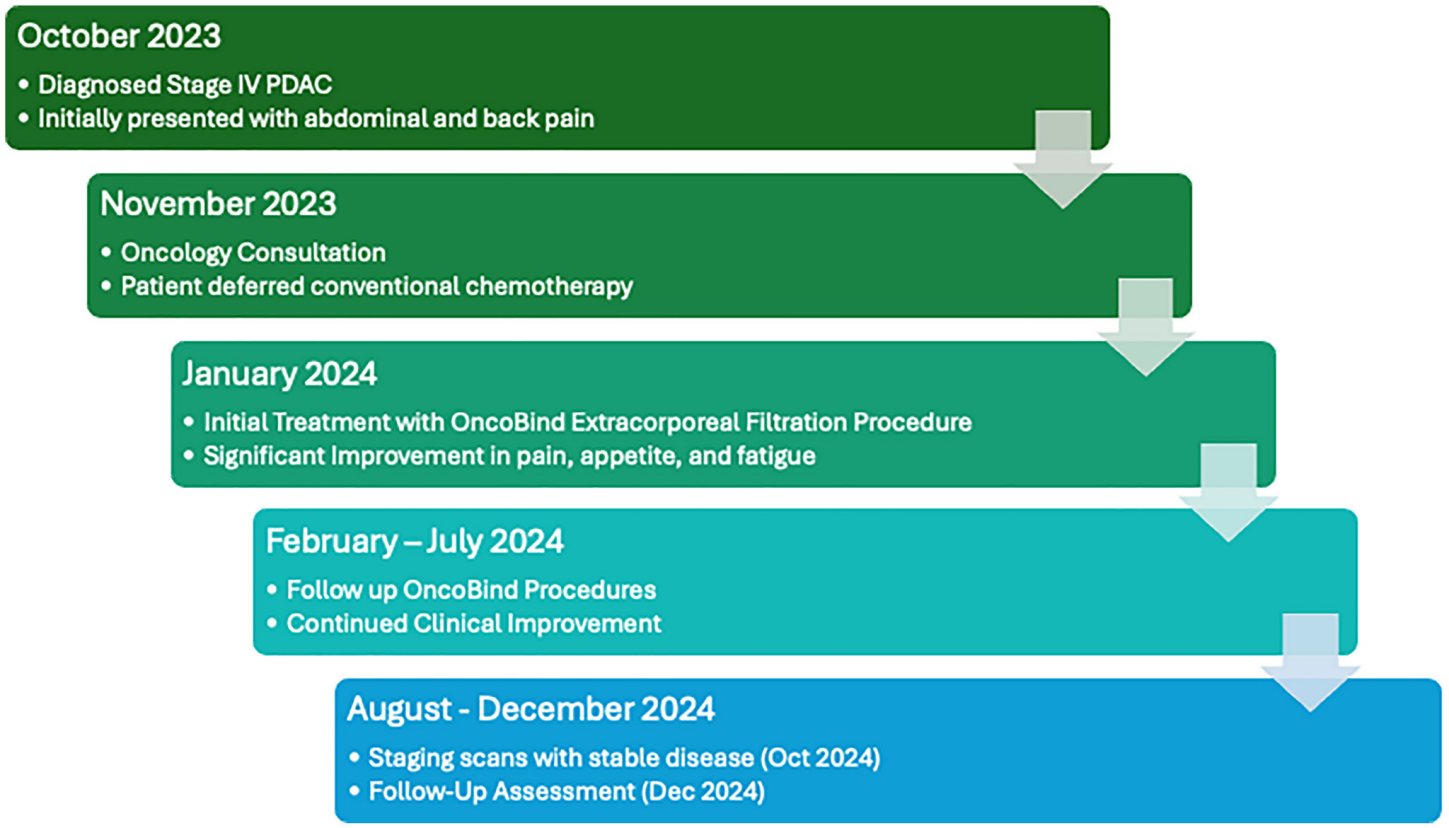
Timeline diagram demonstrating key events.

A 51-year-old woman with hypothyroidism presented with dyspepsia, abdominal pain, and back pain for 1 month. Lab work was significant for elevated White blood cell count at 13,000 cells/μL (reference range 4000–11,000 cells/μL), elevated Alkaline phosphatase at 629 U/L (reference range 35–129 U/L), normal CA-19-9 at 2.4 U/mL (reference range <25 U/mL). Total bilirubin and lipase levels were within normal limits. A CT scan of the abdomen and pelvis revealed a 5.8 cm necrotic pancreatic tail mass invading the spleen along with innumerable liver lesions suggestive of metastatic disease. Numerous porta hepatis and retroperitoneal lymph nodes were noted, with the largest measuring up to 15 mm. A CT chest demonstrated a non-specific 4 mm pulmonary nodule. An endoscopic ultrasound guided biopsy of the pancreatic tail mass revealed poorly differentiated pancreatic adenocarcinoma. Histology revealed malignant tumor cells with marked pleomorphism and anisonucleosis highlighted by BerEp4, cytokeratin seven, and cytokeratin 8/18. In addition, a chemosensitivity test performed by Maintrac lab (Nurnberg, Germany) suggested tumor resistance to standard of therapy. This assessment is based on testing drug effectiveness directly on the cells released by the tumor into the bloodstream and obtained via liquid biopsy. Subsequently, the patient was seen in our oncology clinic and offered several options for standard of care chemotherapy. However, after carefully considering her options, she decided to defer conventional chemotherapy and planned to pursue extracorporeal therapy with the Seraph 100 filter in a country outside the US where the filter is approved for CTC removal. She received her first treatment with Seraph100 filter 3 months after her initial diagnosis. A hemodialysis catheter was inserted into the patient, and the Seraph device was placed in-series with the dialyzer of a 2008T hemodialysis machine (Fresenius, Bad Homburg v. d. Höhe, Germany). Blood flow rate was an average of 200 mL/min and the duration of treatment was up to a maximum of 180 minutes. The patient reported significant improvement in pain and appetite after her first treatment. She received three treatments sessions in one week. Her opioid requirements decreased from Oxycodone 250 mg/day to 25 mg/day. She reported significant improvement in fatigue and quality of life. She was seen in our oncology clinic with significant clinical improvement as evidenced by improved appetite, decreased pain medication requirements, and improved functional status. She subsequently received more sessions at 4, 7 and 12 months after the initial diagnosis. Seraph 100 treatment in the US was performed under an IRB approved clinical trial with informed consent. Seraph 100 treatments conducted outside the US were performed on-label in those jurisdictions. In all follow up procedures, average flow rate was 100–150 ml/min over 120–180 minutes (Figure 1). Post treatment the patient reported feeling well with a good appetite, but 3–4 weeks after an extracorporeal therapy, her fatigue and cachexia would return. Due to travel limitations after her treatment at 7 months, the patient had a long gap between treatments and reported significant fatigue, decreased appetite, and nausea. She reported abdominal swelling from malignant ascites and underwent therapeutic paracentesis. Imaging revealed unchanged size of the pancreatic tail mass and similar size of hepatic and lymph node metastases. There was no evidence of new metastatic disease or progression of disease on scans 1 year post diagnosis (Figure 2). The patient did not report any adverse events associated with the filtration procedure; however, the patient did develop a hematoma from vascular access placement during her first filtration session.

## DISCUSSION

Despite milestone developments in the landscape of cancer diagnosis and management over the past few decades, metastatic disease continues to present a major challenge in terms of cancer morbidity, mortality, and clinical management. Over 65% of cancer deaths in solid tumors are caused by metastatic disease [5]. Metastasis is a complex, multi-step process with various different mechanisms proposed in literature [6]. Circulating tumor cells (CTC’s) are tumor cells that are shed from the primary tumor and travel via blood or lymphatic system to form micro metastases in distant organs under a suitable environment [7]. With the advent of liquid biopsy, there has been significant interest in studying isolation techniques for CTCs and exploring their utility for predicting distant metastasis and disease prognostication [8]. CTCs are precursors to the development of metastases and therefore, there may be therapeutic benefits from high-yield CTC removal in metastatic or potentially metastatic cancer [3]. The Seraph is a single-use filter containing ultra-high molecular weight polyethylene beads that are surface modified with heparin fragments mimicking portions of the endothelial glycocalyx found in the endothelium of mammalian cells [9, 10]. *In vitro* experiments demonstrated Seraph removes circulating tumor cells (CTCs) from blood in a time-dependent fashion [3]. In one study, blood filtration by Seraph 100 resulted in a statistically significant decrease in CTCs across all study patients [4]. Removal of CTCs by extracorporeal filtration could potentially have clinical implications by preventing or delaying metastatic disease and thereby improving patient outcomes. Pain management in metastatic disease is an important consideration in oncologic care and significantly impacts quality of life. Patients with metastatic pancreatic disease often present with very high levels of pain. Interestingly, presence and severity of pain serves as an important prognostic factor in patients with pancreatic cancer [11]. Presence of pain is associated with decreased survival in these patients [12]. Our patient had near complete resolution of pain after 3 sessions as evidenced by a significant reduction in her opioid requirements. While the precise mechanism underlying pain relief through extracorporeal filtration remains unclear, we hypothesize that it may be due to a reduction in circulating tumor cells (CTCs), which may improve tissue microcirculation. Additionally, the reduction of pro-inflammatory cytokines, such as IL-6, IL-10, and IL-16—known mediators of pain—could also contribute to this significant analgesic effect [13]. This hypothesis requires further study.

Most of the experience with Seraph 100 filter has been in patients with infection and in those patients, the Seraph 100 does not appear to remove any plasma substances that are salutary, and the filter does not remove antibiotics significantly [14]. However, studies in cancer patients have not been rigorously conducted and assessments of potential removal of chemotherapy agents have not yet been performed.

To the best of our knowledge, this is the first reported case where removal of CTCs by extracorporeal filtration was associated with significant improvement in pain and stable disease on imaging 1 year from diagnosis of metastatic, poorly differentiated pancreatic adenocarcinoma. Further trials will be required to evaluate if CTC removal results in improved clinical outcomes. Liquid biopsy may be a valuable tool to assess the efficacy of filtration and quantify CTCs. In our patient, there was a significant reduction in CTCs observed after filtration sessions (Table 1). Prospective studies are needed to determine the safety and utility of this novel treatment modality for CTC removal in patients with metastatic disease.

**Table 1:** Circulating tumor cell (CTC) levels for our patient during treatment course with extracorporeal blood filtration

Date	Type of procedure	CTC baseline (N/ml)	CTC 30–45 min after (N/ml)	CTC 120 min after (N/ml)
Jan 2024	Baseline	150		
June 2024	Treatment	100	<10	^*^
Aug 2024	Treatment	50	<10	<10
Sep-2024	Follow-Up	<10		

^*^Specimen inadequate.
